# Environmental Exposures and Gene Regulation in Disease Etiology

**DOI:** 10.1289/ehp.9951

**Published:** 2007-05-21

**Authors:** Thea M. Edwards, John Peterson Myers

**Affiliations:** 1 Department of Zoology, University of Florida, Gainesville, Florida, USA; 2 Environmental Health Sciences, Charlottesville, Virginia, USA

**Keywords:** chemicals, disease risk, DNA methylation, drug resistance, endocrine disruption, environment, fetal origins of adult disease, gene expression, gene regulation, susceptibility

## Abstract

**Objective:**

Health or disease is shaped for all individuals by interactions between their genes and environment. Exactly how the environment changes gene expression and how this can lead to disease are being explored in a fruitful new approach to environmental health research, representative studies of which are reviewed here.

**Data sources:**

We searched Web of Science and references of relevant publications to understand the diversity of gene regulatory mechanisms affected by environmental exposures with disease implications.

**Data synthesis:**

Pharmaceuticals, pesticides, air pollutants, industrial chemicals, heavy metals, hormones, nutrition, and behavior can change gene expression through a broad array of gene regulatory mechanisms. Mechanisms include regulation of gene translocation, histone modifications, DNA methylation, DNA repair, transcription, RNA stability, alternative RNA splicing, protein degradation, gene copy number, and transposon activation. Furthermore, chemically induced changes in gene regulation are associated with serious and complex human diseases, including cancer, diabetes and obesity, infertility, respiratory diseases, allergies, and neurodegenerative disorders such as Parkinson and Alzheimer diseases. One of the best-studied areas of gene regulation is epigenetics, especially DNA methylation. Our examples of environmentally induced changes in DNA methylation are presented in the context of early development, when methylation patterns are initially laid down. This approach highlights the potential role for altered DNA methylation in fetal origins of adult disease and inheritance of acquired genetic change.

**Conclusions:**

The reviewed studies indicate that genetic predisposition for disease is best predicted in the context of environmental exposures. Second, the genetic mechanisms investigated in these studies offer new avenues for risk assessment research. Finally, we are likely to witness dramatic improvements in human health, and reductions in medical costs, if environmental pollution is decreased.

Over the last 20 years, endocrine disruption research has shown how chemicals in our environment can profoundly affect development, growth, maturation, and reproduction by mimicking hormones or interacting with hormone receptors. One important mechanism of endocrine disruption is altered gene expression, mediated by inappropriate activation or deactivation of receptors that act as transcription factors.

Yet, receptor-mediated changes in gene expression are just the tip of the iceberg. There are many more mechanisms of gene regulation that are potentially susceptible to alteration by environmental influences. The effect of environmental contaminants on health is a major concern because exposure is associated with a number of diseases, including cancer, diabetes, and infertility.

The purpose of this review is to identify points of gene expression regulation, occurring along the process described by the central dogma (DNA → RNA → protein), that have been shown to be affected by environmental factors, particularly contaminants (Figure 1). We have drawn on research that shows a strong connection between environmentally induced changes in gene regulatory mechanisms and disease etiology (Figure 1).

## Pesticides, Gene Translocation, and Non-Hodgkin Lymphoma

A number of cancers, including childhood leukemia and follicular non-Hodgkin lymphoma (NHL), are characterized by specific translocations (Smith et al. 2005; Tsujimoto et al. 1985) promoted by physical proximity of the affected genes within the nucleus [reviewed by Verschure (2004)]. In follicular NHL, the anti-apoptotic B-cell leukemia/ lymphoma 2 (*bcl-2)* gene, normally found on chromosome 18, translocates to the immunoglobulin heavy chain locus on chromosome 14 (Roulland et al. 2004; Tsujimoto et al. 1985). This t(14;18) translocation places *bcl-2* under the control of the heavy chain enhancer, resulting in the overexpression of *bcl-2* and, consequently, increased cell survival and lymphomagenesis [reviewed by Roulland et al. (2004)].

The t(14;18) translocation occurs in the lymphocytes of healthy people and those with follicular NHL, and not all people with follicular NHL possess the translocation (Baccarelli et al. 2006; Roulland et al. 2004). Nonetheless, increased frequency of the translocation is a marker for increased lymphoma risk (Schroeder et al. 2001). Epidemiologic evidence indicates a positive association between t(14;18)-positive NHL and exposure to a variety of pesticides, including dieldrin, toxaphene, lindane, atrazine, and fungicides (Schroeder et al. 2001). Roulland et al. (2004) show that occupational exposure to pesticides can increase the frequency of the t(14;18) translocation, both in terms of the number of people affected, and the number of affected lymphocytes within those people. Exposure to 2,3,7,8-tetrachloro-dibenzo-*p*-dioxin (TCDD) is also correlated with increased number of circulating t(14;18) positive lymphocytes (Baccarelli et al. 2006). It appears that t(14;18) translocation frequency depends on how often pesticide exposure occurs, such that NHL risk increases with ongoing accumulation of genetic instability, acquired as a result of pesticide exposure (Roulland et al. 2004). Thus, variability in environmental exposure, coupled with genetic events like translocation, alters disease risk.

## Environmental Factors, DNA Methylation, and Fetal Origins of Adult Disease

A growing number of animal studies show that parental diet and other exposures can influence fetal DNA methylation patterns and permanently affect health outcomes in later life (Dolinoy et al. 2006; Wu G et al. 2004; Yu et al. 1999). Moreover, there is evidence that environmentally induced changes in DNA methylation patterns are heritable across generations (Anway et al. 2005; Newbold et al. 1998, 2000; Ruden et al. 2005; Turusov et al. 1992; Walker and Haven 1997). To understand how and when environmental factors might change DNA methylation, why these changes are potentially heritable, and how they can contribute to the fetal origins of adult disease, it is helpful to consider examples in the context of ontogeny.

### Ontogeny of DNA methylation and environmental influence

DNA methylation occurs in two modes: dynamic methylation and theoretically permanent methylation, such as X chromosome inactivation and genomic imprinting (Bestor 2000). In dynamic methylation, DNA methylation/demethylation reactions turn genes on or off throughout the life of an organism (Cervoni and Szyf 2001). For example, Wilks et al. (1984) showed active demethylation of the vitellogenin promoter in response to estradiol treatment in chickens. The more permanent, although not irreversible, methylation patterns are determined during early embryogenesis (Guerrero-Bosagna et al. 2005; Hajkova et al. 2002; Reik et al. 2001; Tan et al. 1993; Wu G et al. 2004) and continue to adjust through the neonatal period (Day et al. 2002; Weaver et al. 2004).

**Figure 2:**
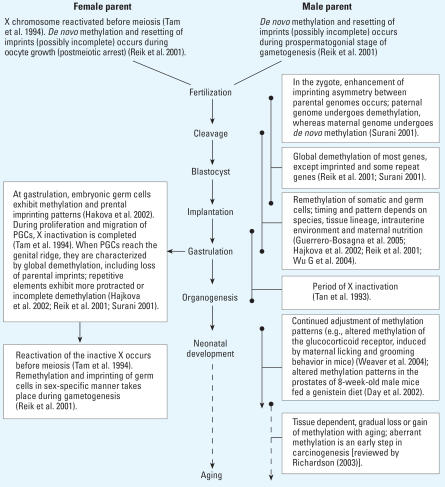
Ontogenetic timeline of methylation in mammals. PGCs, primordial germ cells. From parental gametogenesis, through fertilization, embryonic and neonatal development, and aging, the genome experiences stages of methylation and demethylation. Asymmetry of methylation is sometimes observed between maternal and paternal genomes, between somatic and germ cells, and among different tissues. Environmental factors have been shown to influence methylation patterns at multiple points in development. Please see “Ontogeny of DNA methylation and environmental influence” for details.

Figure 2 shows a developmental timeline for the establishment of methylation patterns. During gametogenesis, each parent resets most but not all the imprints and methylation patterns in his or her germ cells (Reik et al. 2001). This ensures that the parent passes on an imprinted pattern that exclusively reflects his or her own sex. Between fertilization and implantation, the embryo demethylates most of its genes, with the exception of imprinted and some repeat genes (Reik and Walter 2001; Reik et al. 2001; Surani 2001). The maintenance of imprinted genes through the preimplantation period is essential for normal embryonic development (Surani 2001; Wu Q et al. 2004). However, demethylation of other genes is important for making the genome broadly available to the undifferentiated and developing embryo. In addition, demethylation in the embryo may help to remove epigenetic modifications acquired during parental gametogenesis. Between cleavage and gastrulation, remethylation occurs throughout the embryo. X chromosome inactivation occurs over the period between implantation and organogenesis (Tan et al. 1993). At gastrulation, the primordial germ cells (PGCs) are newly formed and exhibit methylation similar to the surrounding somatic cells (Hajkova et al. 2002). Some germ cells also exhibit X inactivation (Tam et al. 1994). During PGC proliferation and migration, X inactivation is completed (Tam et al. 1994). However, when the germ cells enter the genital ridge, their DNA undergoes global demethylation, including loss of parental imprints, and reactivation of the inactive X (Hajkova et al. 2002; Reik et al. 2001; Reik and Walter 2001; Surani 2001; Tam et al. 1994). Whether demethylation affects all genes is under debate. Remethylation and imprinting then occur in a sex-specific manner during gametogenesis (Reik et al. 2001). After birth, somatic cell methylation patterns continue to adjust, based on developmental and environmental factors (Weaver et al. 2004, 2005). As an individual ages, there is a gradual loss or gain of methylation, depending on the cell, tissue, or organ (Ehrlich 2002; Richardson 2003).

Even before conception, organisms are vulnerable to environmentally altered methylation. This phenomenon has been observed in mice exposed preconceptionally to chromium (III) chloride, a carcinogen found in welding fumes. The 10-week-old offspring of male mice treated with chromium 2 weeks before conception exhibit significant increases in serum corticosterone and glucose concentrations (Cheng et al. 2002), as well as increased tumor risk and frequency of developmental abnormalities, relative to controls (Yu et al. 1999). Observed tumors or anomalies include pheochromocytomas, thyroid follicular cell and Harderian gland tumors, ovarian cysts, uterine abnormalities, lung tumors (female offspring only), reproductive gland tumors (male offspring only), and renal nonneoplastic lesions (male offspring only). These effects result, in part, from chromium-induced epigenetic changes in the sperm that alter parental imprinting. Specifically, the sperm of chromium-treated mice exhibit a significant increase in the number of undermethylated copies of the 45S ribosomal RNA gene (*rRNA*) (Cheng et al. 2004; Shiao et al. 2005). *45S rRNA* is the precursor of other rRNAs that are part of the ribosome machinery and a control point for the number of ribosomes in a cell. Increased ribosome number and associated deregulation of protein synthesis could be one step in the progression toward tissue growth, proliferation, and ultimately malignancy (Ruggero and Pandolfi 2003; Shiao et al. 2005).

After conception, between cleavage and gastrulation, the timing and pattern of remethylation is also subject to environmental influence. Amino acid deficiency, for example, causes a marked decrease in overall DNA methylation, along with abnormal expression of the normally silent, paternally imprinted and growth-related *H19* allele in cultured mouse embryos (Doherty et al. 2000). Conversely, Wu Q et al. (2004) showed increased methylation of the *H19*/insulin-like growth factor 2 (*Igf2*) imprint control region, increased methyltransferase activity, and decreased fetal growth after transfer to a recipient dam following *in vitro* exposure of mouse embryos to TCDD from the one-cell stage to the blastocyst stage. TCDD is a widespread and persistent environmental contaminant that is formed during the production of paper, polyvinyl chloride (PVC) plastics and chlorinated pesticides, and during the incineration of chlorine-containing products. Human exposure to TCDD is primarily dietary (Centers for Disease Control and Prevention 2005). Aberrant *H19/Igf2* imprinting and expression are associated with development of a number of tumors, including Wilms tumor (Taniguchi et al. 1995), testicular germ cell tumors (Vangurp et al. 1994), ovarian tumors (Kim et al. 1998), and adrenocortical tumors (Gao et al. 2002). Furthermore, alterations in fetal growth related to *H19/Igf2* imprinting are associated with metabolic disorders in adulthood, including obesity and diabetes (Smith et al. 2006).

In addition to *H19/Igf2* imprinting patterns, other genes that predispose for obesity can be affected by maternal diet. Dolinoy et al. (2006) observed that dietary genistein (the major phytoestrogen in soy) during gestation in mice increased methylation of a retrotransposon located upstream of the *Agouti* gene, effectively reducing expression of the gene. *Agouti* transcription causes yellow fur, obesity, and tumorigenesis. Thus, *Agouti* expression in the unexposed offspring predisposed them to obesity later in life. In addition, only 7% of the genistein-supplemented mice were yellow, compared with 21% of the unsupplemented animals. The degree of DNA methylation was similar in endodermal, mesodermal, and ectodermal tissues, suggesting genistein acts during early embryonic development.

After birth, somatic cell methylation patterns continue to adjust to developmental and environmental factors. Li et al. (1997) showed that neonatal exposure to the synthetic estrogen diethylstilbestrol (DES) caused abnormal demethylation of the CpG sites upstream of the estrogen-response element of the *lactoferrin* promoter. *Lactoferrin* is an important estrogen-inducible glycoprotein component of uterine secretions and is a useful marker of estrogen responsivity (Pentecost and Teng 1987). In another study, Weaver et al. (2004) showed that increased licking, grooming, and arched-back nursing behavior of rat mothers reduced methylation of the glucocorticoid receptor (*GR*) promoter region in the hippocampus. Thus, rats that experienced high-quality (vs. low-quality) maternal behavior exhibited increased *GR* expression, greater glucocorticoid feedback sensitivity, and a better response to stress later in life (lower plasma corticosterone concentrations after 20 min of restraint stress). The epigenetic alteration was noticeable in the first week after birth and persisted into adulthood. The effect could be reversed with cross-fostering, showing it to be a consequence of maternal behavior rather than gestation or genetic inheritance. Furthermore, the effect of high-quality maternal care on *GR* demethylation and reduced stress response could be reversed in adulthood with a central infusion of the methyl donor l-methionine (Weaver et al. 2005). These studies by Weaver et al. (2004, 2005) emphasize the potential for DNA methylation patterns to respond to environmental influences throughout life.

Finally, as an individual ages, there is a gradual loss or gain of methylation, depending on the tissue, cell, or organ. The interaction between aberrant methylation and age is recognized as a possible early step in carcinogenesis [reviewed by Richardson (2003)]. This is especially true of oncogenes or tumor suppressor genes that become incorrectly demethylated or methylated, respectively (Ehrlich 2002). In some cases these changes may be mediated by improperly regulated DNA methyltransferase (Dnmt) enzymes. Gastric cancer cells are often characterized by overexpression of Dnmt1 with hypermethylation of genes relevant to the etiology of gastric cancer, including human MutL homologue 1 (*hMLH1*), thrombospondin-1 (*THBS-1*), and *E-cadherin* (Etoh et al. 2004). This pattern is associated with Epstein-Barr virus infection, which potentially stimulates *Dnmt1* over-expression (Etoh et al. 2004). In other cases, altered methylation of oncogenes or tumor suppressor genes is mediated by dietary folate intake. Folate, found in fresh fruits and vegetables, is needed to make *S*-adenosyl-methionine, the primary methyl donor for methylation (Kraunz et al. 2006). Folate deficiency is associated with hypermethylation of the *p16^INK4A^* (*CDKN2A*) gene in human head and neck squamous cell carcinoma (HNSCC) and a rat model of hepatocellular carcinoma (Kraunz et al. 2006; Pogribny et al. 2004). In HNSCC, degree of *p16* *^INK4A^* silencing is also modified by functional polymorphisms in methylene tetrahydrofolate reductase, an enzyme that regulates serum folate levels (Kraunz et al. 2006).

### Inheritance of methylation patterns

Inheritance of methylation patterns is of great interest because it provides a mechanism by which a parent’s acquired alterations in methylation could be passed to offspring. Anway et al. (2005) showed that gestational exposure of male rats to vinclozolin (anti-androgenic fungicide) or methoxychlor (estrogenic organochlorine insecticide) during the time of gonadal sex determination caused decreased sperm count and viability and increased rates of infertility in adulthood. This loss of fertility was perpetuated through the male germ line for four generations and occurred in conjunction with altered, heritable methylation patterns.

Inheritance of altered methylation patterns could explain the transgenerational effects of DES exposure. DES is a synthetic estrogen given to pregnant women between 1938 and 1971 to prevent miscarriages. The children and grandchildren of humans and mice exposed to DES *in utero* exhibit increased rates of uterine sarcomas and adenocarcinomas, lymphomas, malignant reproductive tract tumors in both males and females, proliferative lesions of the rete testis, and benign ovarian tumors (Newbold et al. 1998, 2000; Turusov et al. 1992; Walker and Haven 1997). Li et al. (2003) showed that DES exposure alters methylation patterns associated with the promoters of many estrogen-responsive genes that regulate reproductive organ development in both mice and humans. In addition, Ruden et al. (2005) recently published a novel hypothesis suggesting that the transgenerational effects of DES are associated with altered DNA methylation, possibly mediated through modified WNT signaling.

## Cadmium, DNA Repair, and Diabetes

In 2003 Schwartz et al. (2003) published a large cross-sectional human study in which they reported a significant positive relationship between urinary cadmium, impaired fasting glucose, and diabetes, suggesting that cadmium exposure plays a role in diabetes etiology. Using monkeys, Kurata et al. (2003) showed that cadmium accumulates in the pancreas and that chronic exposure initiates degeneration of islet β cells and induces the clinical signs of diabetes. Cadmium exposure also potentiates some diabetic complications related to renal tubular and glomerular function (Akesson et al. 2005).

Cadmium-induced diabetes may be a side effect of enhanced DNA repair. A variety of genotoxic environmental factors, including cadmium and several pesticides, cause DNA strand breaks or fragmentation (Bolognesi 2003; Palus et al. 2003; Yang et al. 2003). Poly (ADP-ribose) polymerase-1 (PARP-1) recognizes the strand breaks and promotes break repair by loosening chromatin structure [Benjamin and Gill (1980); reviewed by Rouleau et al. (2004)]. Specifically, PARP-1 catalyzes histone ribosylation—the addition of ADP-ribose molecules to histone lysine residues. The resulting negative charge to the histones causes electrostatic repulsion away from the negatively charged DNA, making it more accessible to repair enzymes (Virág and Szabó 2002). Following DNA repair, the ADP-ribose is freed, becoming briefly available for the stable glycation of other histones and proteins (Cervantes-Laurean et al. 1996; Diefenbach and Bürkle 2005). This mimics glycation caused by hyperglycemia (Gugliucci and Bendayan 1995) and could explain why chronic cadmium exposure induces clinical signs of diabetes.

In addition, sugar-modified histones can undergo other transformations [described by Cervantes-Laurean et al. (1996)] to form advanced glycosylation end products (AGEs). AGE accumulation associated with histones and other proteins is implicated in the progression of aging and age related diseases like diabetes and Alzheimer disease (Cervantes-Laurean et al. 1996; Gugliucci and Bendayan 1995).

## Air Pollution, Activation of Inflammatory Genes, and Respiratory Disease

Respiratory diseases such as chronic obstructive pulmonary disease, cystic fibrosis, interstitial lung disease, acute respiratory distress syndrome, and asthma are characterized by expression of inflammation genes, which can be activated or intensified by exposure to air pollution.

Air pollution consists of tiny ambient particles measuring < 10–15 μm in diameter (PM_10_) that come from dust, smoke, or aerosol liquids produced by vehicles, factories, construction sites, plowed fields, or burning wood. Air pollution can include residual oil fly ash (ROFA), an inorganic mixture of silicates and metal salts containing vanadium, zinc, iron, and nickel released during the combustion of low-grade oil (Henry and Knapp 1980; Samet et al. 2002).

*In vitro* studies with human airway epithelial cells show that exposure to diesel soot and other PM_10_ particles activates pro-inflammatory genes in a process mediated by free radical/oxidative stress mechanisms (Boland et al. 2000; Donaldson et al. 2003; Gilmour et al. 2001, 2003; Marano et al. 2002). The oxidative stress induces pro-inflammation transcription factors such as nuclear factor κB (NF-κB) and activator protein 1 (AP-1) (Donaldson et al. 2003), which in turn promote increased histone acetyltransferase (HAT) activity, histone acetylation, interleukin-8 (IL-8) protein release (IL-8 is a marker of inflammation), and finally, expression of inflammatory genes (Gilmour et al. 2001, 2003).

ROFA exposure stimulates a similar cascade of events. Using a perfused rabbit lung model, Samet et al. (2002) showed that the vanadium component of ROFA can inhibit tyrosine phosphatases, causing phosphorylation of NF-κB and other proinflammation transcription factors, including activating transcription factor 2 (ATF-2), c-Jun, and cAMP response element binding–protein (CREB). Again, this leads to expression of inflammatory genes, potentially exacerbating respiratory distress.

## Environmental Estrogens, Transcription, and Allergies

Bisphenol A (BPA) is a synthetic estrogen that was investigated for use in birth control pills but was instead favored as a plasticizer for use in polycarbonate plastics, dental sealants, and the lining of food cans. Both BPA and 4-nonylphenol (NP), a derived product of nonionic surfactants, have been shown to activate estrogen receptor alpha (ER-α), induce estrogen-dependent gene expression, and stimulate growth in estrogen responsive MCF7 breast cancer cells (Vivacqua et al. 2003).

The effects of these chemicals are not limited to classical estrogen signaling. Lee et al. (2003, 2004) showed that BPA, NP, and 4-*tert*-octylphenol (OP), used widely in detergents and wetting agents, increased the T-cell allergic response, measured as an increase in interleukin-4 (*IL-4*) mRNA levels, in antigen-stimulated mouse T cells. The *IL-4* promoter in mice and humans contains multiple binding sites for a transcription factor called nuclear factor of activated T cells (NF-AT) (Lee et al. 2004). It appears that BPA, OP, and NP enhance IL-4 production by stimulating the calcium-dependent calcineurin signaling pathway. This causes dephosphorylation of cytoplasmic NF-AT with subsequent translocation of the transcription factor to the nucleus. Lee et al. (2003, 2004) suggest that increased NF-AT concentrations in the nucleus up-regulate IL-4 transcription, causing the T-cell allergic response observed with BPA, NP, or OP exposure.

## Environmental Factors, RNA Stability, and Tumor Development or Reproductive Dysfunction

TCDD is a persistent and widespread environmental contaminant that is a potent carcinogen in rodents and a suspected human carcinogen with multiple modes of action. One way that TCDD promotes carcinogenesis is by stabilizing the mRNA of urokinase plasminogen activator (*uPA*), a serine protease that contributes to matrix turnover and growth of tumor cells (Gaido and Maness 1995; Shimba et al. 2000). High *uPA* mRNA concentrations are found in tumors such as hepatocellular carcinoma but not in healthy tissues, and similarly, survival time is inversely related to *uPA* mRNA concentrations (De Petro et al. 1998). In rat liver cells, the TCDD-induced stabilization of *uPA* mRNA is mediated by a 50-kDa cytoplasmic protein (p50) that binds specifically to sites in the 3′ untranslated region of *uPA* mRNA (Shimba et al. 2000). Shimba et al. (2000) showed that p50 is activated rapidly (in 15 min) by TCDD-mediated phosphorylation. They suggest that p50 stabilizes *uPA* mRNA by protecting nuclease cleavage sites from attack.

In a second TCDD study, Minegishi et al. (2003) showed that TCDD exposure reduced the half-life of luteinizing hormone receptor (*LH-R*) mRNA in rat granulosa cells. This could impact steroidogenesis, luteinization, and ovulation by reducing granulosa cell sensitivity to circulating LH. The authors speculate that TCDD affects production or activity of regulatory proteins that destabilize *LH-R* mRNA. For example, TCDD may facilitate the binding of appropriate proteins to the AU (adenylate/uridylate)-rich elements (AREs) of *LH-R*, thus promoting degradation of the mRNA by exosomes.

In addition to uPA, several other proteases are important in tumor development. Among these are the matrix metalloproteinases (MMPs), which promote tumor invasion. Bao et al. (2006) showed that vitamin D (1α, 25-dihydroxyvitamin D_3_) reduced the mRNA stability of metalloproteinase 9 (*MMP-9*) in a human prostate cancer cell line. This, along with other actions of vitamin D, inhibited the invasive ability of the cancer cells.

The role of vitamin D in the prevention of prostate cancer could be applicable in other situations as well. For example, Ritchie et al. (2003) reported that long-term, low-dose exposure to polychlorinated biphenyls (PCBs) could contribute to an increased risk of prostate cancer in the general human population. PCBs are no longer manufactured in the United States, but even after several decades of banning, PCBs remain a persistent environmental contaminant that people encounter mostly through their diet (Ritchie et al. 2005). Given the study by Bao et al. (2006), which suggests that vitamin D has anti-prostate cancer properties, it is interesting to note that PCBs have been shown to reduce serum vitamin D concentrations in rat dams and their offspring (Lilienthal et al. 2000). It is worth testing if PCBs increase cancer risk via changes in vitamin D metabolism or efficacy with regard to processes like reduced *MMP-9* mRNA stability.

## Pesticides, Impaired Protein Degradation, and Parkinson Disease

Parkinson disease (PD) is a neurodegenerative disease that affects more than 1 million people in the United States alone (Sun et al. 2005). It is diagnosed by the presence of intracytoplasmic inclusions in dopaminergic neurons. These inclusions, known as “Lewy bodies,” are composed primarily of α-synuclein protein aggregates (Baba et al. 1998). Lewy body formation also characterizes some forms of dementia and Alzheimer disease (Tu et al. 1998).

PD can occur in families or sporadically and is associated with both genetic and environmental causes (Moore et al. 2005). A familial form of PD involves genomic triplication and consequent overexpression of the α-*synuclein* gene (Singleton et al. 2003). In both familial and sporadic forms of PD, the timely degradation of α-synuclein is inhibited in part by proteasome dysfunction (Mytilineou et al. 2004), an effect that appears to be exacerbated by α*-synuclein* overexpression (Sun et al. 2005).

Using an immortalized rat mesencephalic dopaminergic cell line (N27 cells) transfected with the human α*-synuclein* gene, Sun et al. (2005) showed that exposure to 30 μM dieldrin (an organochlorine pesticide) impaired proteasome function, resulting in a marked increase in α-synuclein positive aggregates. This dose was deduced by the authors to represent the expected human exposure level after 50 years; dieldrin is lipophilic and bioaccumulates significantly in the central nervous system (Fleming et al. 1994). In addition, when α*-synuclein* was overexpressed the dieldrin worked additively with the protein to impair proteasome function and trigger neuronal apoptosis. Sun et al. (2005) argue that exposure to neurotoxic chemicals such as dieldrin increases risk for PD, particularly among individuals predisposed to α-synuclein accumulation for genetic or age-related reasons. Other pesticides, including the herbicide paraquat the fungicide maneb and the insecticide rotenone, are causally linked to α-synuclein accumulation and dopaminergic cell degeneration and apoptosis [reviewed by Meredith et al. (2004)]. Rotenone is so effective that it is used to generate a rodent model for PD (Betarbet et al. 2000).

## Pharmaceuticals, Gene Amplification/Mutation, and Drug Resistance

When faced with death, cells adapt individually and as a population. For example, in *Escherichia coli*, starvation of *lac*^−^ bacteria on lactose medium induces *lac^+^* revertants. The revertants exhibit either gene amplification (20- to 100-fold) of the *lac*^−^ allele, or a compensatory frameshift mutation that randomly produces the *lac^+^* allele in association with wide-spread, stress-induced, hypermutation (Cairns and Foster 1991; Hastings et al. 2004; Hersh et al. 2004). Amplification of the *lac*^−^ allele (increased gene copy number) eases the starvation stimulus because the allele is leaky, conferring 1–2% of the wild-type β-galactosidase activity (Foster 1994). The revertants are apparently produced *de novo* in response to starvation because they appear more rapidly and at higher frequencies than would be predicted by selection-only models [reviewed by Hersh et al. (2004)]. Hence, the phenomenon is termed “adaptive amplification/mutation.”

*E. coli* provide empirical evidence for the ability of cells to enhance their survival in response to environmental pressures through genomic plasticity and adaptation. A major difficulty that affects cancer therapy is the progressive development of drug resistance observed in a subset of patients. As in the *E. coli* example, tumor cells can respond to treatment by amplifying and/or mutating genes that promote their survival. For example, androgen deprivation is a common therapy for prostate cancer. However, some patients respond well initially to the therapy, but then slowly develop resistance resulting in improved tumor growth (Koivisto et al. 1997). Koivisto et al. (1997) report that this pattern is associated with progressive amplification of the androgen receptor (*AR*) gene along with substantially increased *AR* mRNA expression.

A related observation was made by Copur et al. (1995), who showed that continuous exposure of cultured human colon cancer cells to the colon cancer drug 5-fluorouracil (5-FU), causes thymidylate synthase (*TS*) gene amplification and overexpression of the TS protein. Overexpression of TS conferred 5-FU resistance and provides an explanation for the development of fluoropyrimidine chemotherapy resistance among patients with colon cancer (Copur et al. 1995).

In the context of these studies, is it possible that exposure to environmental contaminants could initiate adaptive amplification/ mutation? In 1989, Prody et al. (1989) published a suggestive but isolated study in which they reported *de novo* 100-fold amplification of the silent serum butyrylcholinesterase (*BtChoEase*) gene in a farmer exposed chronically to organophosphate insecticides, which inhibit BtChoEase. The silent *BtChoEase* gene codes for a defective protein that makes homozygous individuals particularly sensitive to organophosphate poisoning. The amplification was not present in the man’s parents but was inherited by his son, indicating that germ cells were also affected.

### Insecticides, alternative RNA splicing, and pesticide resistance

Not all forms of resistance result from gene amplification or compensatory mutation. Glutathione *S*-transferases (GSTs) make up a family of multifunction enzymes that play an important role in detoxification of xenobiotic compounds (Jirajaroenrat et al. 2001). GSTs contribute to insecticide resistance among insects, including mosquitoes (Brown 1986), and to multidrug resistance in tumor cell lines and cancer patients (Hayes and Pulford 1995). In *Anopheles* mosquitoes, one of the GST genes, *adgst1AS1*, codes for (at least) four RNA splice variants that vary in their binding characteristics with regard to permethrin, a pyrethroid insecticide (Jirajaroenrat et al. 2001). Alternative RNA splicing could explain the rapid increase in permethrin resistance, associated with GST upregulation, observed among *Culex* mosquitoes selected for just one or three generations (Xu et al. 2005). It is not known if pyrethroid exposure causes changes in GST activity in humans.

## Environmental Contaminants, Transposons, and Carcinogenesis

Transposon derived sequences account for at least 45% of the human genome, a hefty proportion when compared with the 1% given over to protein coding regions (Jordon et al. 2003). They are of great medical interest for two reasons. First, LINE-1 (L1) retrotransposons, along with the 412 retrotransposon in *Drosophila* and the VL30 retrotransposons in mice and humans, are activated in the normal course of gonad and gamete development, and in fact, may play a regulatory role in these processes (Brookman et al. 1992; Schiff et al. 1991; Trelogan and Martin 1995). Second, retrotransposons and reverse transcriptase (RT) are activated in the tumors of several different cancers [reviewed by Sinibaldi-Vallebona et al. (2006)]. Sinibaldi-Vallebona et al. (2006) report that drug-mediated inhibition of RT activity or silencing of L1 retrotransposons by RNA interference (RNAi) reduces cell growth and stimulates differentiation of cancer cell lines. These observations suggest an active role for retrotransposons in carcinogenesis that could be related to their original developmental role becoming misregulated later in life.

Further, a small amount of evidence suggests that transposons can be activated by environmental contaminants. Transposon activation has been observed in HeLa cells (human cervical cancer cell line) and vascular smooth muscle cells of mice and humans exposed *in vitro* to the carcinogen benzo[*a*]pyrene (Lu et al. 2000; Lu and Ramos 1998; Stribinskis and Ramos 2006) and in the livers of male mummichogs (fish) exposed to pyrene, a common PAH found ubiquitously in the environment and at several estuarine Superfund sites in the United States (Roling et al. 2004). El-Sawy et al. (2005) reported that nickel activates L1 retrotransposition in transfected HeLa cells. In addition, Morales et al. (2002) showed that serum, testosterone, dihydrotestosterone, and a mixture of 17 organochlorine pesticides stimulated transcription of the L1Hs retrotransposon promoter in human choriocarcinoma cells. These examples support the hypothesis that environmentally induced transposon activity could be important in the etiology of cancer and possibly other diseases.

## Conclusions

The studies reviewed in this article show how environmental factors influence a diverse array of molecular mechanisms and consequently alter disease risk. They emphasize the plasticity of the genome and its regulation, providing support for genomic reaction and adaptation in response to environmental stimuli. Further, they provide direct evidence that chemicals placed in the environment by human activity can and do promote disease by altering gene expression. Epigenetic studies in particular provide insights that further our understanding of fetal origins of adult disease, while also offering new research avenues for the investigation of acquired, and potentially heritable, genetic variation and disease susceptibility.

In terms of environmental contamination, these studies show that attention to ontogenetic processes, genetic background, developmental stage, timing and duration of exposure, and the interactions associated with mixtures is critical to quality risk assessment. Further, they address why a given chemical can have multiple modes of action and why sensitivity to chemical exposure varies among individuals. Most important, these studies indicate new ways of thinking about disease etiology, showing that disease risk is best predicted by considering genetic and environmental factors in tandem.

## Figures and Tables

**Figure 1:**
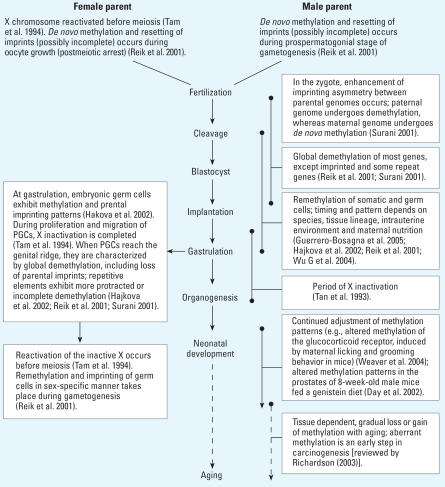
Summary of gene regulatory mechanisms affected by environmental exposures, with disease implications. Abbreviations: BPA, bisphenol A; NP, 4-nonylphenol; PAHs, polycyclic aromatic hydrocarbons, PCBs, polychlorinated biphenlys; OP, 4-*tert*-octylphenol.
